# Extraovarian Brenner tumor in the uterus: a case report and review of literature

**DOI:** 10.1186/s13000-019-0906-1

**Published:** 2020-03-12

**Authors:** Rui-Yue Hu, Yan-Juan Deng, Hao-Hao Zhu, Jing Zhou, Ming Hu, Xiao-Qing Liang, Qiu-Jin Xiao, Lv Zhou, Xiao-Yu Peng, Xing-Wei Zhang, Ning Ji, Huan Deng

**Affiliations:** 1 Department of Pharmacology, Renmin Hospital of Nanchang University, Nanchang, 330006 Jiangxi Province China; 2 Department of Pathology, the Fourth Affiliated Hospital of Nanchang University, 133 South Guangchang Road, Nanchang, 330003 China; 3 Department of Pathology, the 908th Hospital of Chinese People’s Liberation Army Joint Logistic Support Force, Nanchang, 330003 China; 4 Department of Gynecology and Obstetrics, the Fourth Affiliated Hospital of Nanchang University, Nanchang, 330003 Jiangxi Province China; 5 Department of Ultrasonography, the 908th Hospital of Chinese People’s Liberation Army Joint Logistic Support Force, Nanchang, 330003 China; 6 Department of Ultrasonography, the Fourth Affiliated Hospital of Nanchang University, Nanchang, 330003 Jiangxi Province China; 7 Medical College, Nanchang University, Nanchang, 330006 Jiangxi Province China

**Keywords:** Extraovarian, Brenner tumor, Walthard nest, Uterus, Pathogenesis

## Abstract

**Background:**

Extraovarian Brenner tumors (EOBTs) are extremely rare and can be observed incidentally in both female and male patients, raising concerns regarding the origin of Brenner tumors.

**Case presentation:**

A 53-year-old postmenopausal woman presented with a nodular lesion in the left side of the corpus uteri, which was found at a routine health check. Macroscopically, the lesion appeared as a solid nodule with a yellowish-gray cut surface, approximately 6 cm in greatest diameter. Microscopically, the lesion consisted of well-defined epithelial nests and spindled stromal cells. Parenchymal cells expressed CK7, GATA3, CK5/6, 34βE12, and p63. A single layer of cavity-lined cells with umbrella-like shape showed apical Uroplakin III positivity. Stromal cells were positive for SMA, ER, and PR. The final diagnosis was EOBT and the patient was followed for 2 months with no recurrence.

**Conclusions:**

We report here the third case of EOBTs in the uterus. The combination of morphologic and immunohistochemical results supported the involvement of urothelial metaplasia in the development of EOBTs. The similarities between EOBTs and Walthard nests made Müllerian epithelium an attractive candidate as the cellular origin. Changes of tissue structure or sex hormones imbalance may lead to the translocation of Müllerian remnants to distant organs, explaining the pathogenesis of EOBTs.

## Background

Brenner tumors (BTs) are relatively rare and account for approximately 5% of benign ovarian epithelial tumors [1]. It has been recognized that BTs are of benign nature, their most intriguing aspect lies in their histogenesis. The histological features of BTs suggest several cellular origins, including ovarian celomic epithelium, Walthard nests (WNs), mesothelium, Müllerian, and Wolffian cell.

Except for the ovary, it has been reported that BTs may also involve extraovarian tissues. Extraovarian BTs (EOBTs) are extremely rare and mainly occur around the female reproductive system such as the uterus [2, 3], vagina [4–8], broad ligament [9–12], and omentum [13]. Another intriguing clinical observation is that EOBTs were observed occasionally in the testis [14–17] or epididymis [18], further evoking concerns regarding their origins and pathogenesis. However, substantial evidence is limited because of the rarity of EOBTs.

In this study, we report the third case of the uterus BT found beneath the serosa of the left corpus uteri. Immunohistochemistry was used to explore the phenotype of its epithelium and stroma.

## Case presentation

**Fig. 1:**
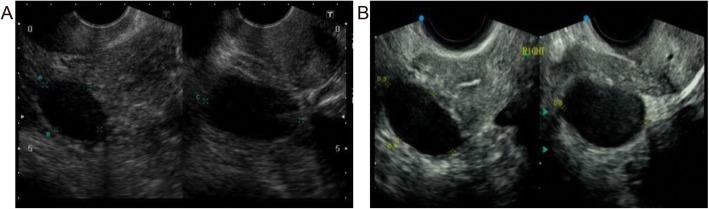
The sonographic findings of the uterine EOBTs. A hypoechoic mass measured 5.2 x 5 x 4.7 cm was detected incidentally 2 years ago (**a**). The lesion was indolent, approximately 5.8 cm in greatest diameter (**b**)

A 53-year-old female patient, gravida 1, para 1, with menopause at age 50, presented to the Department of Gynecology and Obstetrics with a 2-year history of a nodule in the left corpus uteri. The asymptomatic lesion was incidentally detected by ultrasound 2 years ago at a routine health check and had slowly increased in size (Fig. 1a). The sonographic test showed an oval and hypoechoic mass measured 5.8 x 5.7 x 4.6 cm (Fig. 1b). The clinical impression of a leiomyoma was made. Except for an oophorocystectomy of the left ovary 11 years ago for which pathological evaluation revealed a serous cystadenoma, her medical history was silent. Laboratory studies were within normal limits. Biopsy via fractional curettage and colposcope excluded endometrial or cervical-derived tumor. At laparotomy, a nodular neoplasm, protruding into the peritoneal cavity, was located beneath the serosa of the left side of the corpus and completely removed. The patient was followed up for 2 months with no recurrence.

### Histopathology

**Fig. 2:**
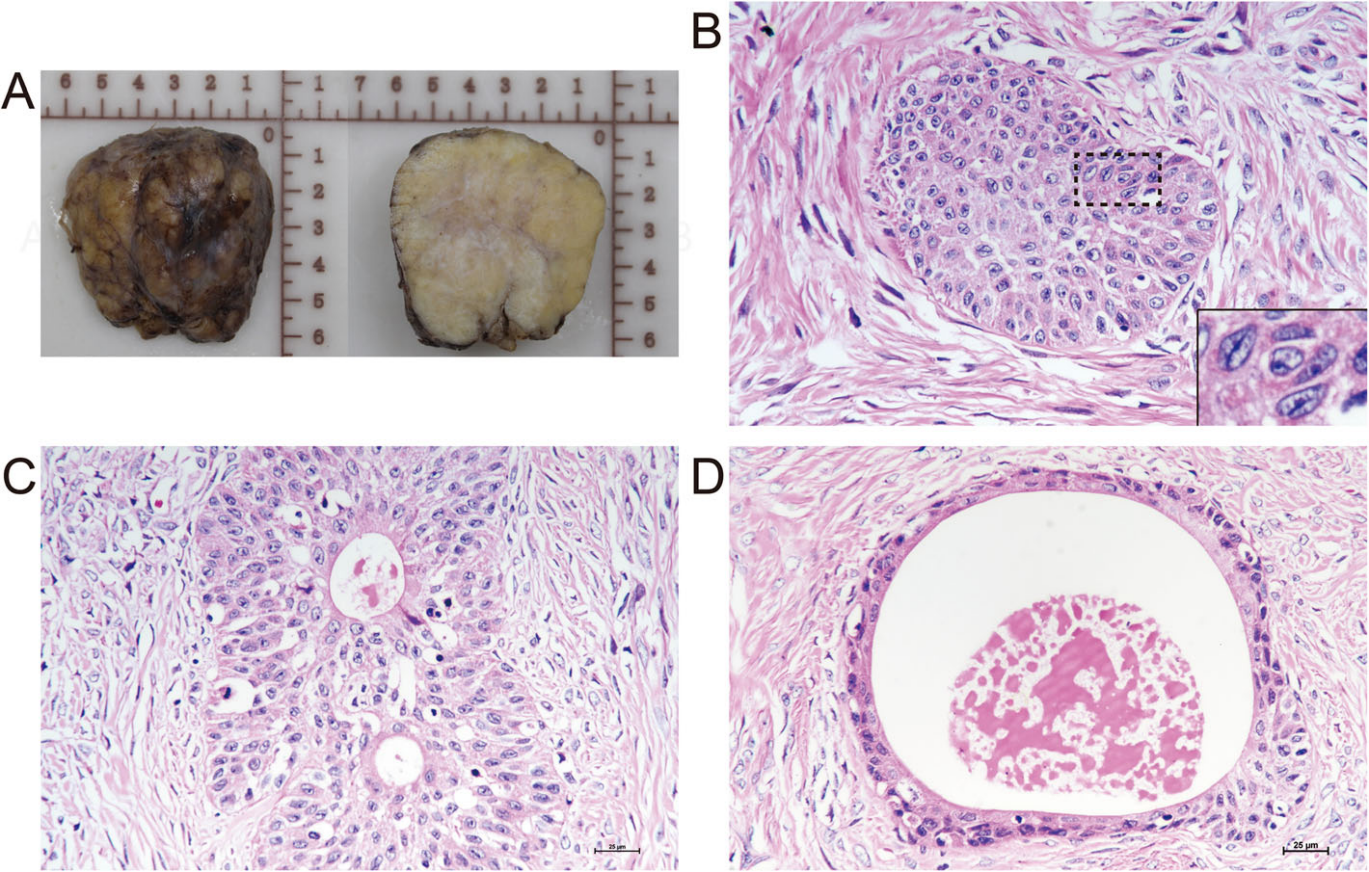
Histopathological features of EOBTs. A nodule lesion with yellowish-gray cut surface (**a**). The well-defined epithelial nests consisted of urothelium-like cells with clear to eosinophilic cytoplasm and longitudinal nuclear grooves (**b**). Cavities in epithelial islands were lined by a single layer of umbrella-like cell (**c**). Some cell nests resembled their counterparts in WNs (**d**). Original magnification x 400

On gross pathological examination, the mass measured 6 x 6 x 5 cm and exhibited a firm and fibrous texture on sectioning. The cut surface was solid and yellowish-gray in color (Fig. 2a). Microscopically, tumor epithelial cells were arranged in oval or cord-like well-defined nests, most of which were surrounded by hyalinized stromal cells (Fig. 2b). Central cavities containing hyaline material were seen in some nests (Fig. 2c). Of note is that a minority of nests consisted of large round cavities that compressed the lining cells resembling WNs (Fig. 2d). Epithelial cells displayed the morphologic continuum from short spindled shapes at the margin of the nests to the umbrella cell-like pattern at the center (Fig. 2c). They contained moderate clear to eosinophilic cytoplasm and oval nuclei with longitudinal grooves (Fig. 2b insert). No intercellular junctions or keratinization were seen. The leading edge of the tumor was examined carefully to explore the direct extension of mesothelium into tumor stroma. Tissue sections showed no evidence of mesothelium-derived cells in the tumor.

### Immunohistochemistry

**Fig. 3:**
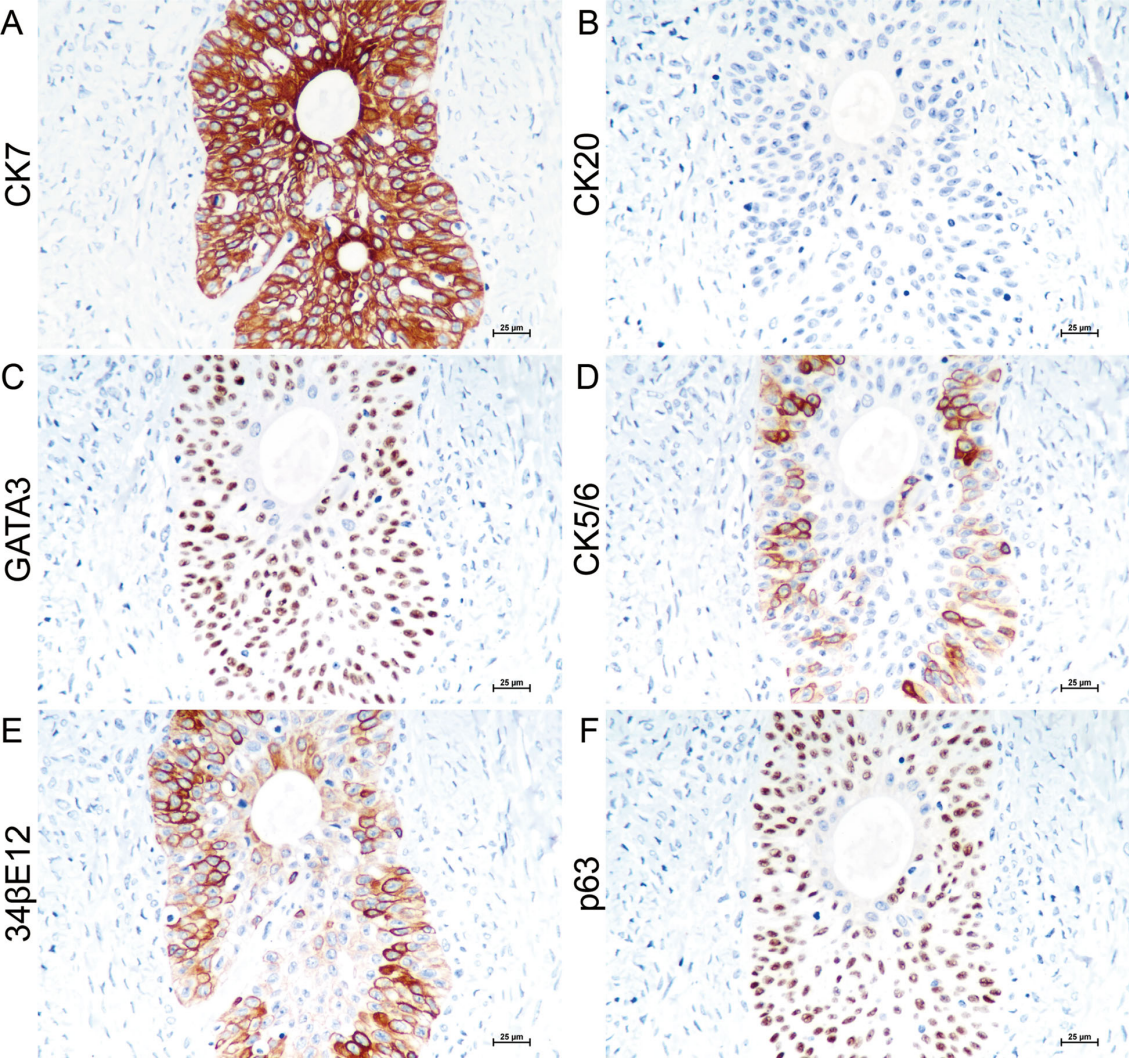
Immunophenotype of EOBTs. Epithelial cells exhibited strong activity for CK7 (**a**), rather than CK20 (**b**). Nuclear positivity for GATA3 (**c**). The graded intensity of CK5/6 (**d**) and 34βE12 staining (**e**) in cell nests. P63 decorated the majority of the epithelium (**f**). Original magnification x 400

Immunohistochemistry (IHC) was performed on serial sections to further confirm the nature of the tumor and explore its pathogenesis. BTs and urothelium-derived tumors commonly show highly overlapping phenotypic features. The primary antibody panel, consisting of cytokeratin (CK) 7, CK20, GATA3, CK5/6, 34βE12, p63, PAX8, p53, p16 and cyclinD1, was used to gain more insights into its nature and whether BT involves the transitional-cells metaplasia. Consistent with previous studies, epithelial cells expressed strong to diffuse reactivity for CK7 and negativity for CK20 (Fig. 3a, b). GATA3 belongs to a zinc finger transcription factor family and plays an essential role in the development of Müllerian duct [19]. It always serves as an important marker to distinguish breast or urinary-derived diseases from their mimics. A strong nuclear immunoreaction was observed for GATA3 in the majority of the epithelial cells (Fig. 3c).

CK5/6 are high-molecular-weight cytokeratins, which decorate basal-type cells in normal urothelium [20]. In this case, epithelial cells displayed a graded intensity for CK5/6 and 34βE12 (Fig. 3d, e). The proportion of 34βE12 positive cells was higher than that of CK5/6 positive cells. P63, an essential factor for the development of urothelial and squamous cells, marks basal and intermediate urothelial cells [20]. Consistent with previous studies, more than 80% of epithelial cells showed nuclear positivity for p63 antibody (Fig. 3f).

**Fig. 4:**
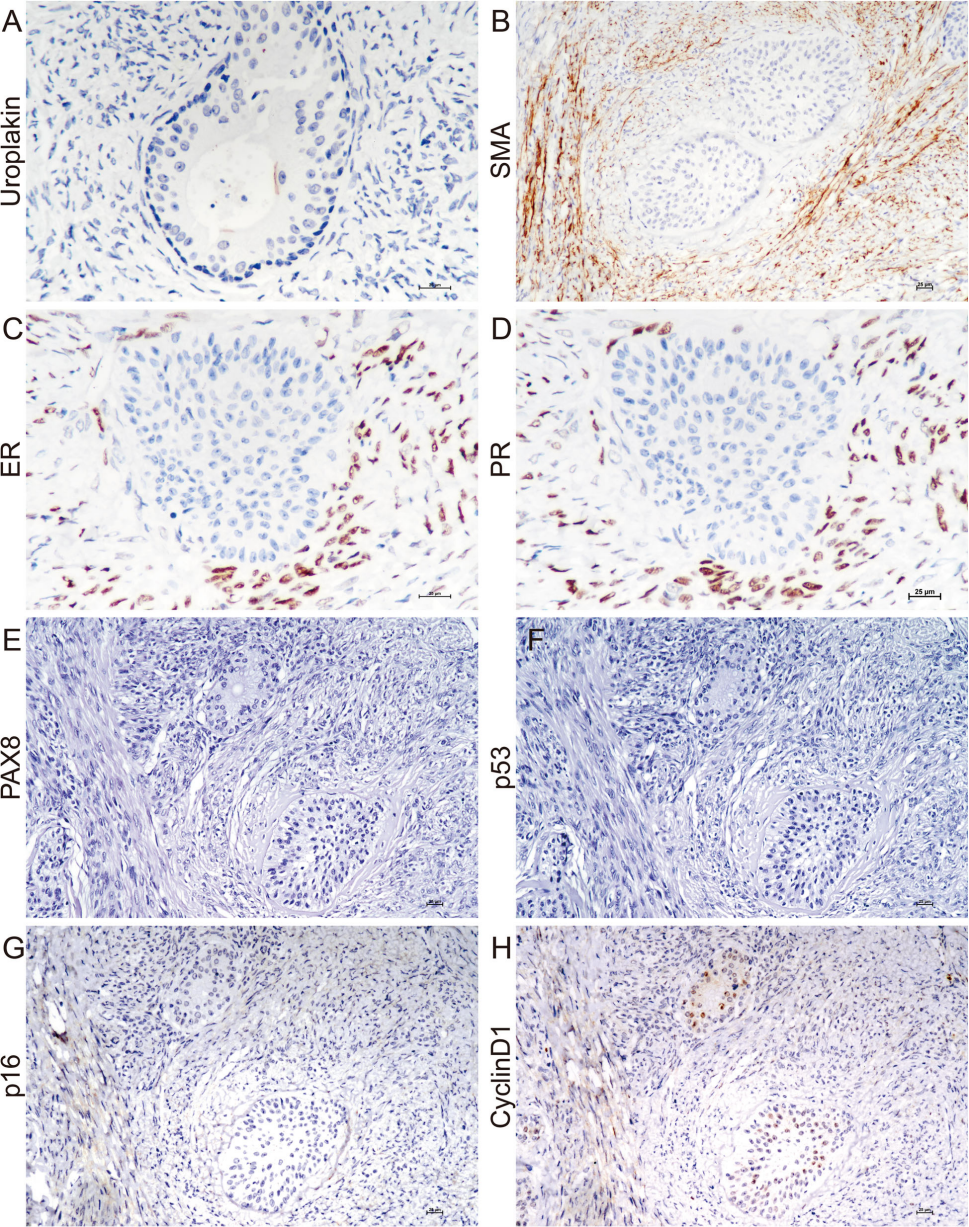
The immunohistochemical results of epithelial and stromal cells. A few of cavity-lined cells exhibited Uroplakin III positivity (**a**). Desmin immunopositivity confirmed smooth muscle nature of stromal cells (**b**). Stromal cells expressed ER (**c**) and PR (**d**). Tumor cells were negative for PAX8, p53, and p16 (**e**-**g**). The epithelium showed weak positivity for cyclinD1 (**h**). Original magnification x 200 (**b**, **e**-**h**) and x 400 (**a**, **c**, **d**)

An interesting observation is that the single layer of cavity-lined cells did not express p63 and CK5/6. However, Uroplakin III revealed a linear apical positivity in a few of these cells (Fig. 4a). Mesothelium markers such as WT1 and calretinin were negative in cell nests (data not shown). The combination of morphologic features and desmin positivity confirmed that spindled stromal cells were smooth muscle cells (Fig. 4b). Like normal counterparts in the corpus uteri, these tumor stromal cells, rather than epithelial cells, expressed estrogen receptor (ER) and progesterone receptor (PR) (Fig. 4c and d). Both epithelial and stromal cells were negative for PAX8, p53, and p16 (Fig. 4e-g). Epithelium showed weak positivity for cyclinD1 (Fig. 4h).

## Discussion

Despite accumulating evidence, the histogenesis of EOBTs remains unsettled. We report the third case of the uterine EOBT and provide preliminary morphologic and immunophenotypic data to further support Müllerian epithelium as an attractive candidate. The clinical and immunohistochemical features of published EOBTs cases from 1947 and our present case were summarized in the Table 1. Studies to date have demonstrated that the fallopian tubes, corpus uteri, and cervix develop from Müllerian ducts. Diffuse activity of epithelial nests for GATA3 supports a Müllerian origin [19]. However, these epithelial cells show negativity for PAX8, a transcription factor involved in the development of Müllerian organs [8, 13, 21]. PAX8 expression can be detected in a small percentage of epithelial cells in WNs. Although the pathogenesis of WNs is largely unknown, it has been suggested that WNs and BTs have analogous origins because of morphologic and immunophenotypic similarities [21]. This view is further supported by the clinical observation that WNs were found in up to 50% of patients with BTs [22]. The epithelial cells of WNs may serve as progenitors of the parenchymal cells of BTs. With regard to the fact that BTs can occur in non- Müllerian-derived tissues, we propose that EOBTs may derive directly from Müllerian remnants or from WNs through a seeding pattern. CK5/6 and p63-expressing basal-like cells located at the edge of EOBTs nests may represent immature cells. Consistent with previous studies, the positivity of Uroplakin III was restricted to the minority of cavity-lining cells, reflecting urothelial metaplasia process [23–25]. The graded intensity of CK5/6 and p63 further supports this possibility.

**Table 1:** Summary of EOBTs

	Sex	Age	Presentation	Location	Suggested origin	IHC	Surgery history
Robinson, 1950 [11]	F	64	Incidental finding	Left broad ligament	Walthard cell nest	n.a.	Negative
Arhelger, 1976 [2]	F	55	Incidental finding	Uterus	coelomic epithelium	n.a.	n.a.
Wagner, 1980 [12]	F	53	Incidental finding	Left broad ligament	coelomic epithelium	n.a.	n.a.
Chen, 1981 [4]	F	67	Incidental finding	Vagina, mid third	Mullerian	n.a.	n.a.
Pschera, 1991 [9]	F	30	Incidental finding	Right broad ligament	Coelomic epithelium	n.a.	n.a.
Hampton, 1992 [10]	F	64	Procidentia	Right broad ligament	Mesothelium	n.a.	n.a.
Rashid, 1995 [5]	F	77	Irritation and soreness	Vagina, not specified	Mullerian or Wolffian	n.a.	Hysterectomy and bilateral salpingo oophorectomy
Ben-Izhak, 1998 [6]	F	68	Incidental finding	Vagina, upper third	Mullerian	Epithelium: ki67 1.0 and 2.5%+, CK(CAM5.2 and AE1/3) + Stromal: vimentin+CK-	Hysterectomy
Ben-Izhak, 1998 [6]	F	72	Vaginal bleeding	Vagina, mid third	Mullerian	Epithelium: ki67 1.0 and 2.5%+, CK(CAM5.2 and AE1/3) + Stromal: vimentin+CK-	n.a.
Angeles-Angeles, 2002 [3]	F	63	Postmenopausal bleeding	Uterus	Mullerian	Epithelium: CK7 + CEA+(weakly)vimentin-thrombomodulin-CD10- Stroma: vimentin+CK7-CEA-CD10-	n.a.
Shaco-Levy, 2013 [7]	F	84	Vulvar irritation and soreness	Vagina, lower third	Mullerian	Epithelium: CK7 + p63 + ER + CK20-PR-vimentin-WT1-Ki67 < 1% + Stroma: vimentin+ER + PR+	Cholecystectomy and bilateral cataract extraction
Hwang, 2017 [13]	F	43	Incidental finding	Omentum	Coelomic epithelium	Epithelium: CKpan+CK7 + GATA3 + UroplainIII+WT1 + p63 + CD34-CD10-CK20-calretinin-ckit-Dog1-Pax8-Stroma: SMA+	n.a.
Park, 2017 [8]	F	76	Incidental finding	Vagina, not specified	Walthard cell nest	Epithelium:GATA3 + p63 + ER + PAX8- Stromal: na	n.a.
This study	F	53	Incidental finding	Uterus	Mullerian	Epithelium:CK7 + GATA3 + 34βE12 +thinsp;CK5/6 + p63 + CK20-UroplakinIII- Stromal cell: SMA + ER + PR+	Oophorocystectomy
Hartz, 1947 [18]	M	38	Hydrocele	Epididymis	Mesothelium	n.a.	n.a.
Vechinski, 1965 [14]	M	67	Swelling of the scrotum	Testis	Nongerminal	n.a.	n.a.
Ross, 1968 [15]	M	61	Incidental finding	Right paratesticular	Wolffian	n.a.	Negative
Goldman, 1970 [16]	M	41	Testicular aching	Left testis	Mullerian	n.a.	Negative
Nogales, 1979 [17]	M	37	Testicular mass	Tunica vaginalis	Coelomic epithelium	n.a.	n.a.

Accumulating evidence subdivides Brenner tumor into benign, borderline, and malignant types [26, 27]. The nature of EOBT is largely unknown. Morphological features of published EOBT cases, including this case, qualify this tumor more as benign BT. However, this concept has been challenged by the molecular signatures. The epithelium expressed p63 and cyclinD1, rather than p53 and p16, reflecting its borderline propensity [13, 26]. Further studies will be required to elucidate the biological details of EOBT.

EOBTs also can be observed in vagina [4–8]. However, the histogenesis of the vagina is under controversy. Previous morphologic evidence supports the traditional concept that the cranial part (upper third) of the vagina originates from the Müllerian duct and the caudal part (lower two-thirds) arises from urogenital sinus. This hypothesis has not yet addressed a critical issue of how the columnar Müllerian epithelium converts to the squamous cell. Genetic studies have raised an intriguing possibility that the whole vagina is derived from the Müllerian duct based on evidence obtained from transgenic models. Knockout of Wnts family members such as Wnt4, 5a, and 9b, which are essential for the formation of Müllerian ducts, resulted in the absence of both the Müllerian ducts and vagina [28–30]. Downregulation of Pax-2, a urogenital transcription factor expressed in the Wolffian and Müllerian ducts, led to the lack of mesonephric and paramesonephric systems, rather than the bladder and urethra [31]. *Hoxa 13*, a critical gene during the development of the caudal Müllerian duct, is highly expressed in the cervix and vagina of newborn rodents [32]. Hoxa 13 can activate promoter activity and upregulate the expression of bone morphogenetic protein 4 (BMP4), which, in turn, enhances p63 level and induces squamous cell conversion [33].

Müllerian remnants have also been detected in the appendix testis and loose connective tissue between the epididymis and testis [34]. Most of reported BT cases in male patients were located in these sites [14–18]. Thus, it is theoretically possible for EOBTs to occur directly in the whole vagina, uterus, fallopian tube, and paratestis. More substantial evidence is needed to further support this view because of the extreme rarity of EOBTs.

Previous studies have failed to reach a conclusion about the role of stromal cells in development of BTs. As the majority of BTs occur in ovary, it is of great interest to explore the link between hormones and stromal cells. Immunohistochemical results showed that the spindled cells were positive for ER and PR in the present study. Although normal smooth muscle cells of the corpus uteri express these receptors in the setting of physical status, stromal cells of vaginal EOBTs also exhibit ER and PR positivity (Table 1), indicating the involvement of sex hormone-related signaling pathways [7]. Like their ovarian counterparts, EOBTs are usually found in postmenopausal women, who have low levels of circulating estrogen and progesterone. However, androgen production does not suffer from menopause because of sparing of the stromal compartment, suggesting that ageing and sex hormones disorder may be risk factors for BTs. Alterations of the microenvironment may trigger the pathogenesis of EOBTs or WNs.

One hypothesis emerges in elucidating how BTs occurs in non-Müllerian-derived ovary. When the fimbria keeps close to the ovary, Müllerian cells seed and implant on the surface of the ovary to form inclusion cysts, which may develop BTs [35]. The mechanisms of how EOBTs happen in tissues far from Müllerian-derived organs are largely unclear. Up to 57% (4/7) EOBTs patients with available history underwent abdominal surgeries (Table 1). We propose that abdominal surgery or laparotomy, which leads to the alteration of tissue structure, can promote the translocation of Müllerian epithelium to distant sites.

## Conclusion

EOBTs are extremely rare and can be found incidentally in both male and female patients. We report the third case of uterine EOBTs with morphologic and immunophenotypic analyses. The well-defined epithelium islands consisted of urothelium-like cells, which exhibited morphologic continuum from basal- to umbrella-like pattern. The reactivity for CK7, GATA3, CK5/6, 34βE12, and Uroplakin III further supported the possibility that these tumor cells were undergoing urothelial metaplasia. The combination of patient’s age at diagnosis (most > 50 years) and ER/PR positivity in stromal cells suggests the involvement of sex hormones imbalance. Overlapping features of WNs and EOBTs implies a common pathogenesis from Müllerian epithelium. Changes of environment such as inflammation and surgery may promote the translocation of Müllerian remnants to distant tissues. Further studies are needed to determine the underlying molecular mechanisms.

## Data Availability

Data sharing is not applicable to this article as no datasets were generated or analyzed during the current study.

## Abbreviations

BTs: Brenner tumors
CK: Cytokeratin
EOBTs: Extraovarian BTs
ER: Estrogen receptor
PR: Progesterone receptor
WNs: Walthard nests
